# Should We Still Focus That Much on Cardiovascular Mortality in End Stage Renal Disease Patients? The CONvective TRAnsport STudy

**DOI:** 10.1371/journal.pone.0061155

**Published:** 2013-04-19

**Authors:** Claire H. den Hoedt, Michiel L. Bots, Muriel P. C. Grooteman, Albert H. A. Mazairac, E. Lars Penne, Neelke C. van der Weerd, Piet M. ter Wee, Menso J. Nubé, Renée Levesque, Peter J. Blankestijn, Marinus A. van den Dorpel, for the CONTRAST investigators

**Affiliations:** 1 Department of Internal Medicine, Maasstad Hospital, Rotterdam, The Netherlands; 2 Department of Nephrology, UMC Utrecht, Utrecht, The Netherlands; 3 Julius Center for Health Sciences and Primary Care, UMC Utrecht, Utrecht, The Netherlands; 4 Department of Nephrology, VU Medical Center, Amsterdam, The Netherlands; 5 Institute for Cardiovascular Research VU Medical Center (ICaR-VU), VU Medical Center, Amsterdam, The Netherlands; 6 Department of Nephrology, Centre Hospitalier de l’Université de Montréal, St-Luc Hospital, Montréal, Canada; University of Louisville, United States of America

## Abstract

**Background:**

We studied the distribution of causes of death in the CONTRAST cohort and compared the proportion of cardiovascular deaths with other populations to answer the question whether cardiovascular mortality is still the principal cause of death in end stage renal disease. In addition, we compared patients who died from the three most common death causes. Finally, we aimed to study factors related to dialysis withdrawal.

**Methods:**

We used data from CONTRAST, a randomized controlled trial in 714 chronic hemodialysis patients comparing the effects of online hemodiafiltration versus low-flux hemodialysis. Causes of death were adjudicated. The distribution of causes of death was compared to that of the Dutch dialysis registry and of the Dutch general population.

**Results:**

In CONTRAST, 231 patients died on treatment. 32% died from cardiovascular disease, 22% due to infection and 23% because of dialysis withdrawal. These proportions were similar to those in the Dutch dialysis registry and the proportional cardiovascular mortality was similar to that of the Dutch general population. cardiovascular death was more common in patients <60 years. Patients who withdrew were older, had more co-morbidity and a lower mental quality of life at baseline. Patients who withdrew had much co-morbidity. 46% died within 5 days after the last dialysis session.

**Conclusions:**

Although the absolute risk of death is much higher, the proportion of cardiovascular deaths in a prevalent end stage renal disease population is similar to that of the general population. In older hemodialysis patients cardiovascular and non-cardiovascular death risk are equally important. Particularly the registration of dialysis withdrawal deserves attention. These findings may be partly limited to the Dutch population.

## Introduction

End-stage renal disease (ESRD) patients have a high cardiovascular (CV) mortality rate, which is substantially higher compared to the general population, especially in young subjects[1]; [2]. The United States (US) renal data system registry reports 43% of deaths to be cardiovascular [3]. However, mortality patterns differ considerably between ESRD patients from the US, Europe and Asia [4]. Differences in patients who start with dialysis, in selection of patients for transplantation and peritoneal dialysis (PD) and in life expectancy between the areas greatly affect mortality rates and cause specific mortality distribution [4]. In the Dutch CONvective TRAnsport STudy (CONTRAST) we found an incidence of CV mortality of 34/1000 person years in the patients treated with online hemodiafiltration (HDF) and 42/1000 person years in the patients treated with low-flux hemodialysis (HD) [5]. Whereas the US HEMO study reported 66 cardiac deaths per 1000 patients years [6] and the ERA-EDTA reported a unstandardized CV mortality rate of 74.9 CV deaths per 1000 persons years for the period 1994–2004 in European patients [7]. The latter study showed that patients starting dialysis have a generally increased risk of death that is not specifically caused by excess CV mortality [7].

The first objective of the present analysis was to compare the distribution of death causes, in particular CV deaths, in the CONTRAST cohort to the Dutch HD population and to the Dutch general population. The second objective was to study potential differences in risk factors between those who died from cardiovascular disease (CVD), from infections or from dialysis withdrawal. The third objective was to assess factors related to dialysis withdrawal.

## Methods

Study design and methods of CONTRAST have been published before [5]; [8]. In short, CONTRAST was a randomized controlled trial comparing effects of online HDF versus low-flux HD on all-cause mortality and CV morbidity and mortality. Patients with end-stage renal disease undergoing chronic intermittent HD for at least two months and aged 18 years or above were enrolled from June 2004 until January 2010 in twenty-nine dialysis centers in The Netherlands (n = 26), Canada (n = 2), and Norway (n = 1). Patients were eligible for inclusion if they were treated two or three times per week with low-flux HD. Exclusion criteria were: treatment with hemo(dia)filtration or high-flux HD in the six months preceding randomization, a life expectancy less than three months due to non-renal disease, participation in another clinical intervention trial evaluating cardiovascular outcomes and severe non-adherence regarding frequency and/or duration of dialysis treatment.

The study was conducted in accordance with the Declaration of Helsinki and approved by the medical ethics review boards of all participating hospitals. Written informed consent was obtained from all patients prior to enrolment.

### Dialysis Procedures

Online HDF was performed in the post-dilution mode, with synthetic high-flux dialyzers [5]. HD patients were treated with synthetic low-flux dialyzers. All patients were treated with ultrapure dialysis fluids, defined as less than 0.1 colony forming units per mL and less than 0.03 endotoxin units per mL. Routine patient care was performed according to national and international Quality of Care Guidelines.

### Data Collection

Standardized forms were used to collect demographical, clinical and laboratory data. Type of vascular access, duration of dialysis (dialysis vintage), medical history (presence of diabetes mellitus (DM) and previous CVD), were recorded. CVD was defined as previous stroke or transient ischemic attack, peripheral vascular disease and/or coronary heart disease (CHD), including history of angina pectoris, myocardial infarction or prior coronary revascularization. Dialysis vintage was determined as the sum of time patients were treated with HD or PD before inclusion. At each three monthly visit, occurrence of clinical events was recorded. Routine blood samples were drawn prior to dialysis. Patients with a urinary production of less than 100 mL per day were considered anuric.

### Health Related Quality of Life 36 (SF-36)

Health related quality of life (HRQOL) was assessed with the validated KDQOL-SF version 1.3 (http://gim.med.ucla.edu/kdqol/downloads/-download.html) [9]; [10], which contains the SF-36 version 1 [11]. The eight domains of the SF-36 were summarized into a physical functioning (Physical Component Summary – PCS) and a mental functioning (Mental Component Summary – MCS) score. These summaries are constructed so that a score of 50 represents the mean of the general United States population with a standard deviation of 10 [12]. A difference of 3 points in the summary score has been proposed to be clinically relevant [11]; [12].

### Laboratory Measurements

Routine measurements were done in the participating hospitals using standard techniques. Serum albumin was measured in local hospitals with the bromcresol green method or bromcresol purple method and samples that were measured with the bromcresol purple method were converted to bromcresol green concentrations with the formula: bromcresol green = bromcresol purple+5.5 [13]. Inflammatory markers from 405 patients were measured centrally. hsCRP (mg/L) was measured with a particle-enhanced immunoturbidimetric assay on a Roche-Hitachi analyzer (Roche Diagnostics GmbH, Mannheim, Germany), with a lower quantification limit of 0.1 mg/L. IL-6 (pg/mL) was measured with an ELISA (Sanquin, Amsterdam, The Netherlands), with a lower quantification limit of 0.35 pg/mL.

### Outcome

Cardiovascular deaths were defined as death from CV causes, which included myocardial infarction, ischemic or haemorrhagic stroke, sudden death (unexpected death within an hour of symptom onset or unwitnessed, unexpected death without obvious non-cardiac cause in patients known to be well within the past 24 hours), heart failure or other CV causes. Non-CV causes were defined as death from infections, cancer, dialysis withdrawal, unnatural deaths and other non CV causes, such as gastrointestinal haemorrhage or respiratory insufficiency. If no documentation on the event was available, death was categorized as unknown cause. An independent Endpoint Adjudication Committee reviewed source documentation (mostly discharge letters from admissions to the hospital) for all deaths, to adjudicate the cause of death using their clinical experience. Each case was reviewed by three or, if necessary, four medical doctors and the cause of death was defined when two doctors agreed. We made no priory definition to adjudicate deaths as ‘dialysis withdrawal’. For patients whose cause of death was adjudicated as dialysis withdrawal, we retrieved source documentation and explored circumstances and the interval between dialysis withdrawal and death.

The present analyses were restricted to patients who died either during treatment with HD or HDF or who died within 28 days after censoring due to transplantation, switch to PD, switch to other hospital or stopping because of other reasons. The survivor group consisted of patients who remained on HD or HDF during the study up to the end of follow-up, or up to censoring.

### Comparison CONTRAST with Other Registries

#### Renine

Renine is the nation-wide Dutch registry for HD patients with a 100% coverage [14]. We used data from the year 2008, because patients in CONTRAST were included between 2004–2010. Cause of death was coded using the ERA-EDTA codes [15]. Code 11 denotes acute myocardial infarction, code 22 strokes and codes 12 and 15 sudden death. Codes 14, 16 and 18 denote heart failure and codes 13, 26 and 29 other CV causes. We used code 30–39 for infections, code 66 and 67 for cancer and code 51, 53 and 61 for withdrawal from HD. Code 17, 21, 23–25, 27–28, 41–44, 46, 62–64, 69–73, 90 and 102 were categorized as other non CV death. Code 52 and 80–82 were categorized as unnatural event and code 99 and 0 and ‘cause of death not yet registered’ were classified as unknown cause of death.

#### General population of the Netherlands

Causes of death of the general population were obtained at the central office of statistics of the Netherlands [16]. Eurostat ICD codes were used to classify causes of death in 2008 [17]. To determine the number of deaths from a CVD cause we used the number from ‘total deaths from CV diseases’ (ICD-10 I00-I99) minus the numbers of deaths caused by pulmonary embolism or other diseases of pulmonary origin (I26–28), or by infectious causes of heart disease, such as endocarditis (I33, 38–41), or by diseases from veins, arterioles, capillaries and lymph vessels I78–89 and by hypotension (I95).

### Data Analysis

#### Comparison CONTRAST with the Dutch general population

We multiplied the 5 year age and sex specific mortality rates from the Dutch population with the age and sex specific number of person years in CONTRAST to obtain an estimate of the number of CV deaths and total deaths expected, had the Dutch mortality rate be applicable for CONTRAST. Next we summed the age and sex specific expected numbers of death to obtain an overall number of CV and total deaths. Next, the proportion of expected CV death/total expected death was estimated to come up with a proportion CV death in CONTRAST, had the general population mortality rates been applicable for the CONTRAST population.

#### Baseline characteristics of patients in CONTRAST by cause of death

To compare the baseline characteristics of the patients by cause of death, we ran multivariable regression models, adjusted for age and sex. To compare medication use between the different causes of death, we additionally adjusted for history of CVD. We performed Chi square test to explore differences in causes of death by the age groups younger than 60, 60–75 and older than 75 years. Two sided *P*-values below 0.05 were considered statistically significant. These analyses were conducted with SPSS software (version 18.0; SPSS Inc. Headquarters, Chicago, Illinois, US).

## Results

Baseline characteristics are given in Table 1.

**Table 1:** Baseline characteristics of the CONTRAST participants.

Variable	CONTRAST participants = 714
Age (years)	64.1±13.7
Male sex - no. (%)	445 (62)
Region	
- Netherlands – no. (%)	597 (84)
- Canada- no. (%)	102 (14)
- Norway – no. (%)	15 (14)
History of cardiovascular disease – no. (%)	313 (44)
Diabetes mellitus – no. (%)	170 (24)
Dialysis vintage (years)	2 (1–4)
Systolic blood pressure (mmHg)∼	147±21
Diastolic blood pressure (mmHg)∼	75±12
Body weight (kg)	72.4±14.4
BMI after dialysis (kg/m^2^)	25.4±4.8
Residual kidney function – no. (%)*	376 (53)
eGFR (ml/min/1.73 m^2^)	3.2 (1.3–5.5)
Treatment frequency	
3x/week – no. (%)	668 (94)
−2x/week – no. (%)	44 (6)
Duration of a dialysis session – min	226±23
Bloodflow (mL/min)	308±39
Dialysis access – no. (%)	
-Fistula	567 (80)
-Graft	100 (14)
-Central catheter	47 (6)
spKt/V_urea_	1.40±0.22
Hemoglobin (mmol/L)	7.3±0.78
Phosphorus – mmol/L	1.64±0.49
Beta-2-microglobulin – mg/L	31.5±14
Albumin (g/L)∧	40.4±3.8
Creatinine (µmol/L), pre-dialysis	861±255
C-reactive protein mg/L (n = 405)	3.9 (1.4–10.4)
Interleukin-6 pg/mL (n = 403)	2.1 (1.2–3.8)
***Quality of life***	
Physical Composite score	39±11
Mental Composite score	50±12
***Prescribed medication – no. of patients(%)***	
Beta blocker	53
Alfa blocker	10
RAAS inhibitor	49
Statin	51
Platelet aggregation inhibitor	31

Values are means ±SD, median (interquartile range) or number (percentage).

∼ pre-dialysis.

* residual kidney function if diuresis >100 ml/24 h.

∧albumin concentrations measured with the bromcresolpurple method have been converted to the bromcresolgreen method.

eGFR = estimated glomerular filtration rate; RAAS = renine angiontensin aldosterone system.

To convert hemoglobin in mmol/L to g/dL divide by 0.62; phosphorus in mmol/L to mg/dL, divide by 0.323; albumin in g/L to g/dL, divide by 10; creatinine in µmol/L to mg/dL divide by 88.4.

### Causes of Death in CONTRAST

Two hundred thirty one patients died. Seventy-four patients (32%) died from CV causes and 142 patients (62%) died from non CV causes. Infections (22% of all deaths) and dialysis withdrawal (23% of all deaths) were the main non CV causes of death. In 7% of cases the cause of death was unknown (Table 2).

**Table 2:** Causes of death in CONTRAST and Renine.

Cause of Death	CONTRAST deaths			Renine deaths of 2008¥			
*Cardiovascular*	n	%	95% CI of %	n	%		95% CI of %
Acute Myocardial Infarction	8	3.5	(1.1–5.8)	73	6.5		(5.0–7.9)
Cerebrovascular Accident∧	6	2.6	(0.5–4.6)	41	3.6		(2.5–4.7)
Sudden death	37	16.0	(11.3–20.7)	105	9.3		(7.6–11.0)
Heart failure	11	4.8	(2.0–7.5)	62	5.5		(4.2–6.8)
Other cardiovascular	12	5.2	(2.3–8.1)	27	2.4		(1.5–3.3)
**Total cardiovascular**	**74**	**32.0**	**(26.0–38.0)**	**308**	**27.2**		**(24.6–29.8)**
***Non cardiovascular***							
Infection	51	22.1	(16.7–27.4)	180		15.9	(13.8–18.0)
Cancer	14	6.1	(3.0–9.1)	81		7.2	(5.7–8.7)
Unnatural death	3	1.3	(0–2.8)	8		0.7	(0.2–1.2)
Dialysis withdrawal	54	23.4	(17.9–28.8)	217		19.2	(16.9–21.5)
Other non cardiovascular	20	8.7	(5.0–12.3)	59		5.2	(3.9–6.5)
**Total non cardiovascular**	**142**	**61.5**	**(55.2–67.7)**	**545**		**51.0**	**(45.2–51.1)**
Unknown	15	6.5	(3.3–9.7)	247		21.8	(19.4–24.2)
Miscellaneous	n.a.	n.a.	n.a.	32		2.8	(1.9–3.8)
***Total number of deaths***	***231***	***100***		***1132***		***100***	

¥ Renine population January 1st 2008: 4921 hemodialysis patients, mean age 64.4 years, 59.7% male.

∧Cerebrovascular accident ischemic or hemorrhagic.

n.a. = not applicable.

### Death Causes in Renine

Renine comprised 4921 patients at the first of January 2008, with a mean age of 64.4 years and 59.7% men. 27% of deaths was of CV origin. Sudden death was the cause of death in 9% of cases. Sixteen percent of patients died from an infection and 19% withdrew from dialysis treatment. In 25% the death cause was unknown (Table 2).

### The Percentage CV Death in CONTRAST when Dutch General Population Rates Apply

The expected number of CV- deaths in CONTRAST when the general population mortality rates were applied was 10.2; 7.7 in men and 2.5 in women. The expected number of all cause death was 35.5; 26.4 in men and 9.2 in women. The contribution of CV-death to all-cause death would therefore be 27.8% (95% CI 13.1–42.4%) based on general population mortality rates. In CONTRAST the proportion of CV-death was 32.0% (95% CI 26.0–38.0%). (Table 3).

**Table 3:** Comparison of cardiovascular and non cardiovascular death rates of CONTRAST patients and the Dutch general population.

	Dutch general population					CONTRAST			Standardized G.P.	
Age category	People at risk	CV deaths n	Total deaths	Rate of CV death/personyear^a^	Rate of all causedeath/personyear^b^	Personyears lived^c^	CV deaths n	Total deaths	Expected CVD deaths (n)^d^	Expected total deaths (n)^e^
**Men**										
40–44	660 196	177	897	0.00026810	0.00135868	53.00	1	2	0.01420	0.07201
45–49	631 041	333	1379	0.00052769	0.00218527	66.03	3	3	0.03484	0.14429
50–54	573 890	530	2222	0.00092352	0.00387182	79.74	1	3	0.07364	0.30873
55–59	549 639	792	3404	0.00144094	0.00619315	89.82	2	10	0.12942	0.55626
60–64	499 340	1219	5119	0.00244122	0.01025153	117.47	8	17	0.28677	1.20424
65–69	356 075	1494	5937	0.00419574	0.01667345	109.29	9	19	0.45855	1.82224
70–74	274 517	2130	7812	0.00775908	0.02845725	185.03	10	36	1.43566	5.26544
75–79	206 572	3180	10614	0.01539414	0.05138160	149.88	8	41	2.30727	7.70107
80–84	125 720	3567	11335	0.02837257	0.09016067	69.64	6	21	1.97586	6.27878
85–89	57 780	2964	9008	0.05129802	0.15590169	17.50	2	7	0.89771	2.72827
90–94	15 560	1292	4012	0.08303341	0.25784061	1.07	0	1	0.08884	0.27588
**Total**	**3950330**	**17678**	**61739**			**938.47**	**50**	**160**	**7.7028**	**26.3573**
**Women**										
40–44	642 708	79	643	0.00012291	0.00100045	33.11	0	0	0.00406	0.03312
45–49	621 972	154	1143	0.00024760	0.00183770	45.24	0	0	0.01120	0.08313
50–54	567 963	211	1697	0.00037150	0.00298787	52.39	3	4	0.01946	0.15653
55–59	540 372	284	2412	0.00052556	0.00446359	47.31	2	5	0.02486	0.21117
60–64	495 939	503	3424	0.00101423	0.00690407	50.59	3	8	0.05131	0.34927
65–69	369 384	667	3692	0.00180570	0.00999501	94.83	5	14	0.17123	0.94782
70–74	314 518	1235	5185	0.00392664	0.01648554	97.57	1	11	0.38312	1.60849
75–79	278 271	2407	8231	0.00864984	0.02957907	86.76	7	19	0.75046	2.56628
80–84	216 243	4168	12480	0.01927461	0.05771285	43.76	3	10	0.84345	2.52551
85–89	132 311	5338	14778	0.04034434	0.11169139	6.24	0	0	0.25174	0.69695
90–94	51 771	3791	10478	0.07322632	0.20239130	0	0	0	0	0
**Total**	**4231452**	**18846**	**64163**			**557.8**	**24**	**71**	**2.5109**	**9.1783**

a calculated by the total number of people that died of cardiovascular disease (CVD) in the general population in 2008 divided by total number of people at risk.

b calculated by the total number of people that died in the general population in 2008 divided by total number of people at risk.

c number of personyears lived during CONTRAST.

d rate of cardiovascular (CV) death in the general population multiplied by the number of person years lived in CONTRAST.

e rate of all cause death in the general population multiplied by the number of person years lived in CONTRAST.

G.P. = general population.

### Baseline Characteristics of Patients in CONTRAST by Cause of Death

**Figure 1:**
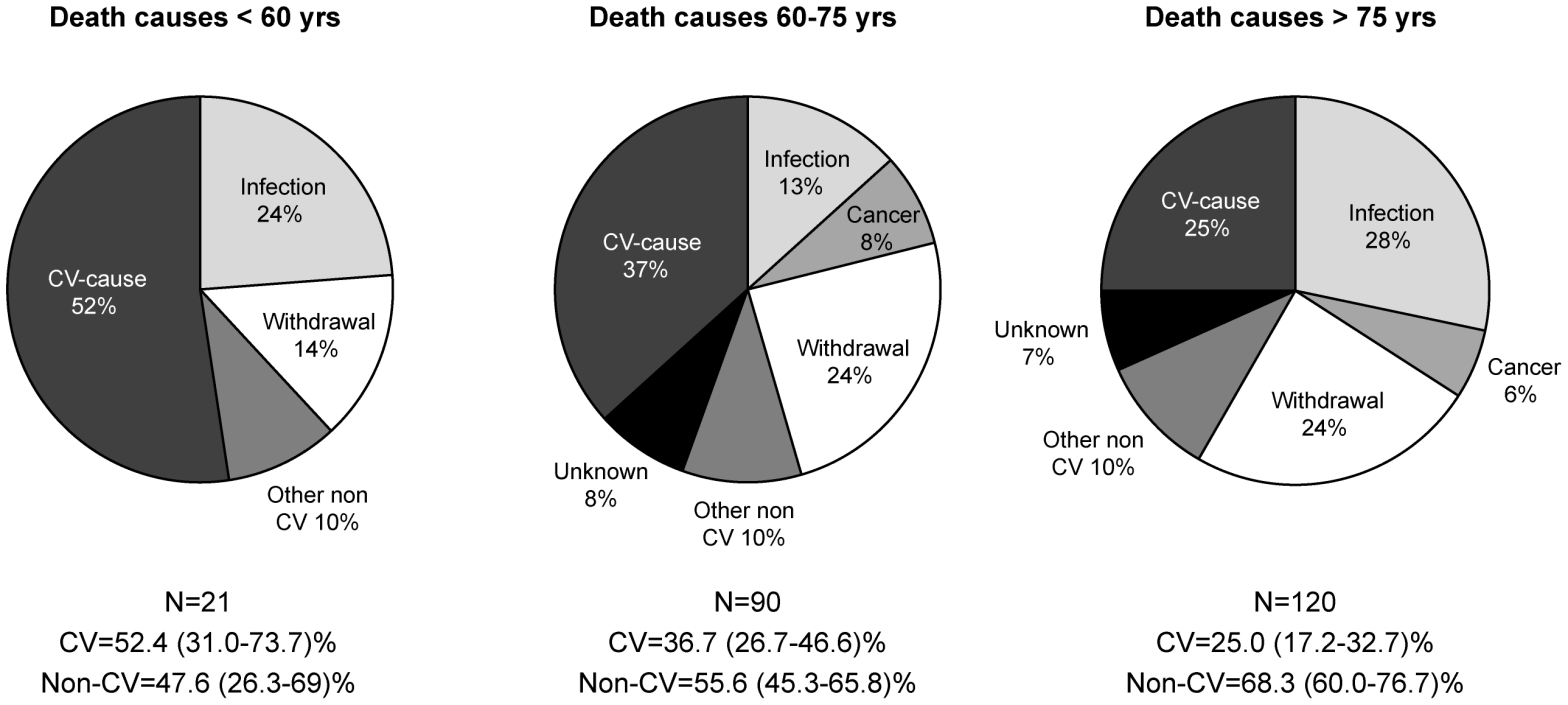
The distribution of death causes across age groups at time of death.

Patients that died from CVD were younger (age at time of death: 71.1 (IQR 65.9–78.9)) than patients dying from infections (76.9 (70.3–81.4)) or after dialysis withdrawal (76.4 (69.2–80.6) (Table 4). The proportion of CV death was larger in patients younger than 60 years old than in patients aged 60–75 and older than 75 years (Figure 1, *P* = 0.02).

**Table 4:** Baseline characteristics of patients who died during follow-up, from cardiovascular death, infection or dialysis withdrawal and of patients who survived (on treatment analysis).

Characteristics	Cardiovascular death (n = 74)		Fatal infection (n = 51)		Dialysis withdrawal (n = 54)		Survivors (n = 483)	
	Mean/Median	SD or IQR	Mean/Median	SD or IQR	Mean/Median	SD or IQR	Mean/Median	SD or IQR
***Demographic***								
**Age at randomisation (yrs)**	69.4∧	10	72.9∧	10	72.9∧	8	60.4	14
**Age at time of death**	71.3	10	75.1	10	74.7	8	n.a.	
**Sex (% man)**	67.6		68.6		66.7		59.0	
**Systolic BP (mmHg)**	150	21	146	25	146	21	147	21
**Diastolic BP (mmHg)**	74	11	74	13	71	11	77	12
**History of CVD (%)**	66.2¶∧		47.1#		64.8¶∧		36.0	
**AMI**	23.3¶		10		22.2		11.4	
**CHD**	47.3¶∧		32		48.2∧		23.2	
**Stroke/TIA**	19.4		16		27.8∧		12.5	
**PAD**	21.6		23.5		29.6∧		11.8	
**DM (%)**	27.4		22#		41.5¶∧		23.1	
**BMI (kg/m^2^)**	24.9	4	25.8	6	25.7	6	25.5	5
**Smoking (%yes)**	25.4		16		13.5		19.5	
**RKF (%yes)**	48.7		41.2∧		46.3∧		54.5	
***Dialysis related***								
**Dialysis vintage at randomization (yrs)**	1.6	(1.1–3.7)	2.8	(1.0–5.7)	2.9	(1–4.5)	1.9	(1.0–3.9)
**Total dialysis vintage at death** * **(yrs)**	3.9	(2.3–5.8)	4.1	(2.4–6.1)	4.7	(3.2–6.1)	4.6	(3.0–6.3)
**Vascular access (%)**								
**AV-fistula**	81.1		68.6		75.9		81.0	
**CVC**	4.1		3.9		11.1		6.4	
**Graft**	13.5¶		27.5#∧		13.0		12.0	
***Laboratory***								
**Hemoglobin (mmol/L)**	7.3	0.7	7.4	0.8	7.3	0.8	7.3	0.8
**Albumin (g/L)**	39∧	4	40	4	39∧	4	41	4
**Creatinin (µmol/L)**	792	244	794	220	742∧	231	902	257
**Calcium (mmol/L)**	2.3	0.2	2.3	0.1	2.3	0.2	2.3	0.2
**Phosphorus (mmol/L)**	1.6	0.4	1.6	0.5	1.6	0.5	1.7	0.5
**Parathyroid hormone (pmol/L)**	22.1	(3.9–33)	16.3	(9–32)	17.5	(10–31)	21.9	(11–38)
**B2-microglobulin (mg/L)**	30.6¶	14	35.9∧	15	30.7	13	31.3	14
**Hs-CRP (mg/L)**	8.9∧n =	(2.3–17.1)	5.7∧n =	(3.2–14.2)	9.3∧	(4.2–25.2)	3.0	(1.0–7.3)
**IL-6 (pg/mL)**	2.5∧n =	(1.9–4.0)	3.7∧	(2.5–4.5)	3.6∧	(1.5–8.7)	1.7	(1.1–2.9)
***Quality of Life***								
**SF-36 PCS**	37∧	11	37∧	11	33∧	8	42	10
**SF-35 MCS**	52#	11	51∧	11	50∧	16	53	11
***Medication (% yes)***								
**Calcium antagonist**	39#		36#		15		32	
**Beta blocker**	62#		52		41∧		52	
**RAAS blocker**	47.3		34∧		35∧		53.3	
**Alfa blocker**	9		6		2		11	
**Statin**	58#		42∧		39∧		52	
**Platelet aggregation inhibitor**	42		34		37		29	

n.a. = not applicable; CVD = cardiovascular disease; AMI = acute myocardial infarction; CHD = coronary heart disease; TIA = transient ischemic accident; PAD = peripheral artery disease; DM = diabetes mellitus; BMI = body mass index; RKF = residual kidney function; AV = arterio-venous; CVC = central venous catheter; CRP = C- reactive protein;

IL-6 = interleukin-6; PCS = physical composite score; MCS = mental composite score; RAAS = renin angiotensin aldosterone system.

∧significantly different as compared to survivors, after adjustment for age and sex.

# significantly different as compared to deaths due to dialysis withdrawal, after adjustment for age and sex.

¶ significantly different as compared to deaths due to infection, after adjustment for age and sex.

* total vintage at time of end of study or date of drop-out for survivors.

After adjustment for age and sex, patients dying from CVD had a significantly higher prevalence of previous CVD and CHD compared to patients dying from infections. Patients who died from infection were more likely to have a graft as vascular access than the patients who died from CVD or dialysis withdrawal. They also had higher β2-microglobulin (β2m) levels at baseline. Patients, who died after dialysis withdrawal, had the most co-morbidity at baseline, namely more often a history of CVD, more specifically previous CHD, cerebral vascular disease or peripheral arterial disease. Also their prevalence of DM was higher. Their mental quality of life at baseline, as reflected by the MCS, was significantly worse than in survivors.

### Exploration of those who died after Dialysis Withdrawal

Dying of patients after dialysis withdrawal (n = 54) was often preceded by a (long) hospital stay. Three factors associated with dialysis withdrawal were identified: acute complications, chronic complications and low quality of life. In 18 patients dialysis was stopped in a period in which an acute complication occurred: in 11 patients an infection was present or suspected, 7 patients had peripheral vascular disease with a need for intervention and 7 patients had a gastrointestinal or shunt bleeding. Some patients suffered from combined complications. In 25 patients dialysis was stopped because of chronic complications: in 4 patients cancer was suspected, in others cachexia, dementia, poor general condition, pain, dyspnea, hypotension (in 3 patients due to aorta-valve stenosis) and infections were associated with dialysis withdrawal. Eleven patients withdrew from dialysis because of a low quality of life. The median number of days between withdrawal and death was 6 (IQR 2–9)(these data were available for 39 patients). 46% died within 5 days after the last dialysis session.

## Discussion

In this study we found that in ESRD patients approximately one third of deaths was of CV origin. The proportion of CV death was comparable with that in Dutch registry of dialysis patients (Renine), and in the general population. The contribution of CV death was larger in younger patients. The other main causes of death were withdrawal from dialysis (23%) and infection (22%). Patients, who withdrew from dialysis, had considerable co-morbidity.

Compared to other studies in dialysis patients the proportion CVD deaths in CONTRAST was low [3]; [18]; [19]. Our study seems representative for the Dutch situation, given the comparability with death causes registered in Renine. The European ERA-EDTA registry reported a CV death proportion of 39% [7]. In the 4D study and in the United States Renal Data System (USRDS) CV deaths accounted for approximately 50% of all deaths [3]; [18]. In DOPPS III, CV death ranged from 29% in patients older than 75 years in Australia and New Zealand to 41% in patients aged 45–75 years in Europe and 56% in patients younger than 45 years in North America [19]. The discrepancies between our study and US studies are larger than between our study and other European studies. The differences between the US and the Netherlands may be a result of differences in patient selection for HD and transplantation, as well as differences in clinical practice.

Importantly, the proportion of CV deaths was not different from what would have been expected using the general population estimates. This is in agreement with the EDTA registry data, showing that CV and non CV death are similarly distributed among incident dialysis patients and the general population [7]. As in CONTRAST, they showed that in patients older than 65 years, the excess non CV death was higher than the excess CV death. Similar data were found in DOPPS III [19]. Our data might underestimate CV mortality, since only prevalent patients were included and there are data showing high rates of CV mortality in the first year of dialysis [20]–[22]. However, mortality in the first year of dialysis is greatly dependent on the characteristics of the patients who are selected to start dialysis and pre-dialysis care they have received [20].

Except for age, no large differences in baseline characteristics were found between the patients dying from CVD, infection or withdrawal. The higher prevalence of grafts in patients dying from infections is in agreement with studies showing a higher incidence of vascular access related infections in patients with synthetic grafts as compared to native fistulas [23]; [24]. Furthermore, higher β2m levels associated with infectious mortality were reported in the HEMO study [25].

Withdrawal from dialysis was much more frequent than anticipated. The death attributed to dialysis withdrawal varies highly among studies. Among elderly patients in DOPPS the proportion of death due to dialysis withdrawal varied between 1–4% in Europe, to a maximum of 8% in North America and 14% in Australia and New Zealand [19]. The proportion reported in other studies from the US varied from 6 to 22% [26]; [27].and the European ERA-EDTA reported a proportion of 5.2%, which became 9.1% when the category ‘Suicide/refusal of treatment’ was added [7]. The 19% in Renine and the 20% in a French study were in agreement with our study [28]. The large proportion of patients dying from dialysis withdrawal was not limited to the Dutch patients in our trial, suggesting that withdrawal is not only an issue in a country where euthanasia has been legalized.

The varying percentage of patients dying after withdrawal of dialysis treatment probably reflects differences in registration practices. So far death due to dialysis withdrawal is poorly defined. Dialysis withdrawal will not always be the cause of death when dialysis is discontinued before death [29]. Notably, in case of severe co-morbidity the co-morbid conditions rather than the dialysis withdrawal itself may be the cause of death, especially when patients die within 3 days after a decision to stop dialysis is made [29].

In our study, deaths were adjudicated as ‘withdrawal from dialysis’, if two or three out of three members of the adjudication committee agreed on this. However, at the start of the study, no clear cut criteria were given to define this aspect. Our findings with respect to determinants of ‘death due to dialysis withdrawal’ were similar to that reported by a study from the US identifying old age and chronic diseases as risk factors for dialysis withdrawal [26]. Data from DOPPS III and the ERA-EDTA registry also showed that the proportion of patients who stopped, increased with older age [7]; [19]. Although patients who withdrew from dialysis had much baseline co-morbidity in common, the factors associated to withdrawal from dialysis were quite heterogeneous. CVD was often one of the acute or chronic reasons to decide to stop HD treatment. However, a broad scala of disease conditions was present at the time of dialysis withdrawal. Until now little attention has been paid to dialysis withdrawal in the nephrology community. In the US written policies about appropriate initiation and withdrawal from dialysis have been made [30]. Patients who refuse dialysis, who are terminally ill or permanently unconscious are patients for whom dialysis is inappropriate [30]. Before these guidelines existed, fifteen percent of nephrologists reported in a questionnaire to have written policies on dialysis withdrawal, after these guidelines, still only a minority (30) of nephrologists reported to have such a written policy [31].

Although the young dialysis patients still have a high CV risk, the strong emphasis on the risk of CV death in dialysis patients might be less justified in today’s European dialysis populations [32]. Non CV causes of death and particularly withdrawal from dialysis deserve more attention, particularly in the elderly.

In view of the importance of withdrawal from dialysis in daily practice, well-defined criteria for the registration of dialysis withdrawal are warranted. We would like to propose to define death due to dialysis withdrawal as ‘death occurring more than 5 days after the last dialysis treatment which has been assigned as such by patient and doctor’. A clear definition of dialysis withdrawal is a needed to investigate the incidence and risk factors for dialysis withdrawal. Mostly combinations of several co-morbidities exist, such as CVD, infections, malnutrition, and low quality of life. Regarding malnutrition, we previously showed that nutritional status relates to quality of life [33], whereas reaching clinical performance targets does not [34]. Possibly, less focus on biochemical outcome targets for patients at high risk for dialysis withdrawal, such as elderly dialysis patients might lead to a better quality of life [35]. Potentially, less dietary restrictions and les medication prescriptions might improve nutritional status. Potentially treatable symptoms in hemodialysis, such as bone pain, insomnia and emotional symptoms are undertreated, and if they are treated, then mostly by primary care providers [36]. These kinds of symptoms deserve more attention, since they are part of patients’ quality of life.

Strengths of the present study are the availability of many patient characteristics, which were collected in a standardized way and second the adjudication process for causes of death by an adjudication committee. However, a limitation in the adjudication process was the lack of criteria to adjudicate deaths as ‘dialysis withdrawal’, which might have led to an overestimation. Using our proposed definition would probably lead to a lower percentage of dialysis withdrawal, however we do not have data to make this distinction retrospectively and therefore used the death causes as adjudicated by the committee.

Another limitation is that we did not have individual patient records from patients registered in Renine, therefore we were not able to adjust for potential differences in age and sex distribution between Renine and CONTRAST. Finally, the generalizability to other populations may be considered as a limitation, since the study population mainly consisted of Dutch patients.

In conclusion, although the absolute risk of death is much higher, the proportion of CV deaths in a prevalent ESRD population is similar to that of the general population. In older HD patients CV and non-CV death risk are equally important. Particularly the registration of dialysis withdrawal deserves attention.

## Supporting Information

Appendix S1 Membership of the CONTRAST investigators.
(DOCX)
