# Supplementary material for: Should We Still Focus That Much on Cardiovascular Mortality in End Stage Renal Disease Patients? The CONvective TRAnsport STudy
Source: PLoS One. 2013 Apr 19;8(4):e61155. doi: 10.1371/journal.pone.0061155 (PMC3631204; doi:10.1371/journal.pone.0061155)
Supplement: Appendix S1 — Membership of the CONTRAST investigators. (DOCX) [file pone.0061155.s001.docx]

The following committee members and investigators participated in the CONTRAST-study:
Executive Committee: University Medical Center Utrecht - PJ Blankestijn (co-chair); VU University Medical Center, Amsterdam - MPC Grooteman, MJ Nubé, PM ter Wee (co- chair), Julius Center for Health Sciences and Primary Care, University Medical Center Utrecht - ML Bots; Maasstad Hospital, Rotterdam - MA van den Dorpel
Research physicians: EL Penne; NC van der Weerd; AHA Mazairac; CH den Hoedt

End-Points Committee: Medical Center Alkmaar, Alkmaar - AER Arnold, W Bronsveld, F Stam; University Medical Center Utrecht, Utrecht - WH Boer, PA Doevendans, LJ Kappelle, FLJ Visseren ; VU University Medical Center, Amsterdam - AJ Kooter, YM Smulders, MC Visser, G Veen; College voor Zorgverzekeringen, Diemen: G Ligtenberg

Data and Safety Monitoring Board: Julius Center for Health Sciences and Primary Care, Utrecht - I van der Tweel, Leiden University Medical Center, Leiden - TJ Rabelink; Maastricht University Medical Center, Maastricht - CDA Stehouwer.

Investigators - Canada: Georges-L Dumont Regional Hospital, Moncton - M Dorval; CHUM St Luc Hospital, Montréal - R Lévesque; The Netherlands: Academic Medical Center, Amsterdam - MG Koopman; Catharina Hospital, Eindhoven - CJAM Konings; Dialysis Clinic Noord, Beilen - WP Haanstra; Dianet Dialysis Centers, Utrecht - M Kooistra and B van Jaarsveld; Fransiscus Hospital, Roosendaal - T Noordzij; Gelderse Vallei Hospital, Ede - GW Feith; Haga Hospital, The Hague - M van Buren; Isala Clinics, Zwolle - JJG Offerman; Jeroen Bosch Hospital, 's Hertogenbosch - EK Hoogeveen; Maasland Hospital, Sittard - F de Heer; Maasstad Hospital, Rotterdam - PJ van de Ven; Martini Hospital, Groningen - TK Kremer Hovinga; Medical Center Alkmaar, Alkmaar - WA Bax; Onze Lieve Vrouwe Gasthuis, Amsterdam - JO Groeneveld; Oosterschelde Hospital, Goes - ATJ Lavrijssen; Rijnland Hospital, Leiderdorp - AM Schrander-Van der Meer; Rijnstate Hospital, Arnhem - LJM Reichert; Slingeland Hospital, Doetinchem - J Huussen; St Elisabeth Hospital, Tilburg - PL Rensma; St Fransiscus Gasthuis, Rotterdam - Y Schrama; University Medical Center St Radboud, Nijmegen -HW van Hamersvelt; University Medical Center Utrecht, Utrecht - WH Boer; VieCuri Medical Center, Venlo - WH van Kuijk; VU University Medical Center, Amsterdam - MG Vervloet; Zeeuws-Vlaanderen Hospital, Terneuzen - IMPMJ Wauters. Norway: Haukeland University Hospital, Bergen - I Sekse.
